# Supplementary material for: Dysgu: efficient structural variant calling using short or long reads
Source: Nucleic Acids Res. 2022 Jan 31;50(9):e53. doi: 10.1093/nar/gkac039 (PMC9122538; doi:10.1093/nar/gkac039)
Supplement: gkac039_Supplemental_File [file gkac039_supplemental_file.pdf]

Dysgu: efficient structural variant calling using short or long reads

Kez Cleal[1*], Duncan M. Baird[1]

[1] Division of Cancer and Genetics, School of Medicine, Cardiff University, Heath Park, Cardiff, CF14 4XN, UK.

* To whom correspondence should be addressed: clealk@cardiff.ac.uk; Correspondence may also be addressed to Duncan M Baird: bairddm@cardiff.ac.uk

Supplementary figures and tables

Table of Contents

Supplementary figures and tables ***1***

Table S1. Comparison of benchmarking using svbench and truvari. **4**

Figure S1. SV calling using simulated data. **4**

Table S2. Genotype calling performance. **5**

Illumina paired-end reads ***5***

Table S3. 20× Illumina aligned with bwa mem, All-regions benchmark. **5**

Figure S2. Performance of dysgu using 40× PE reads. **6**

Table S4. 40× Illumina aligned with bwa mem, Tier1 benchmark. **6**

Table S5. 40× Illumina aligned with bwa mem, All-regions benchmark. **7**

Table S6. 40× Illumina aligned with bwa mem, All-regions benchmark, split by size. **7**

Figure S3. Precision and recall of Syndip benchmark, Illumina reads 45×. **8**

Table S7. Syndip benchmark results, Illumina reads 45×. **8**

Table S8. Syndip benchmark, Illumina reads 45×, split by size. **8**

Figure S4. 1000 Genomes benchmark, deletions ≥ 1000 bp. **9**

Table S9. 1000 Genomes benchmark, deletions ≥ 1000 bp. **10**

PacBio Sequel II reads *10*
Figure S5. Performance of dysgu on 8× PacBio reads aligned using ngmlr. **10**
Table S10. 8X PacBio Sequel II reads 8× aligned using ngmlr, Tier1 benchmark. **11**
Table S11. 8× PacBio Sequel II aligned using minimap2, All-regions benchmark. **11**
Table S12. 8× PacBio Sequel II aligned using ngmlr, All-regions benchmark. **11**
Table S13. 8× PacBio Sequel II reads aligned with ngmlr, All-regions benchmark, split by size. **12**
Figure S6. Performance of dysgu on PacBio reads at 15× coverage. **12**
Table S14. 15× PacBio Sequel II reads aligned using minimap2, Tier1 benchmark. **13**
Table S15. 15× PacBio Sequel II reads aligned using ngmlr, Tier1 benchmark. **13**
Table S16. 15× PacBio Sequel II reads aligned using minimap2, All-regions benchmark. **13**
Table S17. 15× PacBio Sequel II reads aligned using ngmlr, All-regions benchmark. **13**
Table S18. 15× PacBio Sequel II reads aligned with minimap2, All-regions benchmark, split by size. **14**
Table S19. 15× PacBio Sequel II reads aligned with ngmlr, All-regions benchmark, split by size. **14**
Figure S7. Precision and recall of Syndip benchmark, PacBio reads 12×. **15**
Table S20. Syndip benchmark results, PacBio reads 12×. **15**
Table S21. Syndip benchmark, PacBio reads 12×, split by size. **15**
ONT reads *16*
Figure S8. Performance of dysgu using ONT reads. **16**
Table S22. 13× ONT Promethion reads aligned using minimap2, Tier1 benchmark. **16**
Table S23. 13× ONT Promethion reads aligned using ngmlr, Tier1 benchmark. **17**
Table S24. 13× ONT Promethion reads aligned using minimap2, All-regions benchmark. **17**
Table S25. 13× ONT Promethion reads aligned using ngmlr, All-regions benchmark. **17**
Table S26. 13× ONT Promethion reads aligned with minimap2, All-regions benchmark, split by size. **18**
Table S27. 13× ONT Promethion reads aligned with ngmlr, All-regions benchmark, split by size. **18**
Combinations of sequencing platforms *19*
Table S28. Combinations of sequencing platforms, Tier1 benchmark. **19**
Table S29. Combinations of sequencing platforms, Tier1+2 benchmark. **19**
Figure S9. Differences in deletion calls between short and long reads using dysgu. **20**
Figure S10. Examples of deletion SVs called correctly using PE reads but not LR. **21**
Table S30. Resource requirements of dysgu using PE data. **22**

Caller	Truvari (274.1 s) Precision	Recall	F1	GT F1	Svbench (28.9 s) Precision	Recall	F1	GT F1
dysgu	0.944	0.409	0.570	0.515	0.955	0.405	0.569	0.517
manta	0.948	0.323	0.482	0.471	0.948	0.330	0.489	0.478
delly	0.857	0.227	0.359	0.354	0.839	0.232	0.363	0.358
lumpy	0.904	0.207	0.337	-	0.888	0.211	0.341	-

Table S1. Comparison of benchmarking using svbench and truvari.

Svbench benchmarking was compared to truvari by testing on 20× coverage PE data, using Tier1 regions as input. Truvari was run using parameters "-P 0 -s 50 --sizemax 260000000 -S 50 -r 1000 --pctsim 0", and svbench was run using parameters "allow_duplicate_tp=False, slop=1000, pcts=0, min_size=50". Numbers in parentheses are the total time taken to run the benchmark for all samples.

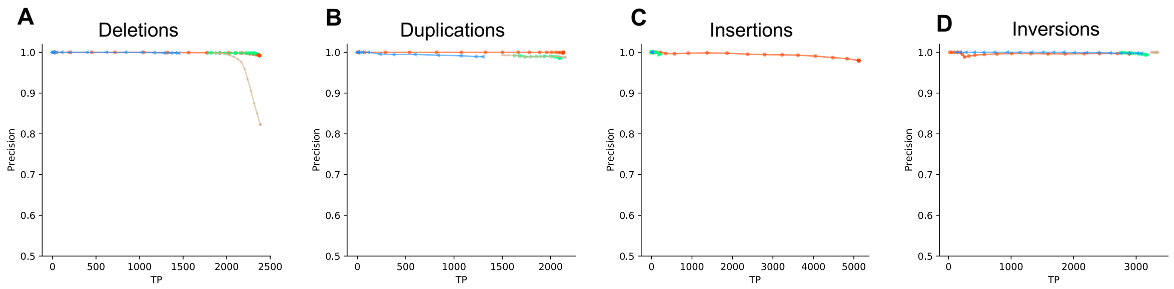

E

SV type	Caller	TP	Precision	Recall	F1
Deletion	dysgu	2375	0.9925	0.2375	0.3833
	manta	1447	0.9979	0.1447	0.2527
	delly	2341	0.9974	0.2341	0.3792
	lumpy	2387	0.8223	0.2387	0.37
Duplication	dysgu	2132	0.9995	0.2132	0.3514
	manta	1297	0.9893	0.1297	0.2293
	delly	2106	0.9855	0.2106	0.347
	lumpy	2152	0.9881	0.2152	0.3534
Insertions	dysgu	5119	0.9799	0.256	0.4059
	manta	4	1	0.0002	0.0004
	delly	218	0.9954	0.0109	0.0216
	lumpy				
Inversion	dysgu	2891	0.9972	0.2891	0.4482
	manta	3081	0.9974	0.3081	0.4708
	delly	3180	0.9938	0.318	0.4818
	lumpy	3345	1	0.3345	0.5013

Figure S1. SV calling using simulated data.

SV callers were tested on simulated 150 bp paired-end data at 25× coverage.

Dataset	SV	Tool	Precision	Recall	F1
20x PE bwa-mem	Deletions	dysgu	0.894	0.606	0.722
		manta	0.920	0.568	0.702
		delly	0.824	0.513	0.632
		lumpy			
	Insertions	dysgu	0.723	0.192	0.304
		manta	0.912	0.134	0.234
		delly	0.897	0.010	0.019
		lumpy			
PacBio 8x minimap2	Deletions	dysgu	0.913	0.921	0.917
		nanovar	0.812	0.872	0.841
		sniffles	0.679	0.721	0.700
		svim	0.737	0.893	0.808
	Insertions	dysgu	0.832	0.903	0.866
		nanovar	0.833	0.843	0.838
		sniffles	0.712	0.823	0.764
		svim	0.675	0.862	0.757
ONT 15x minimap2	Deletions	dysgu	0.844	0.922	0.881
		nanovar	0.875	0.846	0.860
		sniffles	0.534	0.877	0.664
		svim	0.642	0.946	0.765
	Insertions	dysgu	0.852	0.869	0.860
		nanovar	0.879	0.848	0.863
		sniffles	0.657	0.725	0.689
		svim	0.440	0.879	0.587

Table S2. Genotype calling performance.

Callers were tested against Tier1 regions using the HG002 benchmark dataset. The read-type, coverage values and mapper used are shown in the left-hand column.

Illumina paired-end reads

	TP		FP		Precision		Recall		Duplication		F1	
	DEL	INS	DEL	INS	DEL	INS	DEL	INS	DEL	INS	DEL	INS
dysgu	6902	4841	206	193	0.971	0.962	0.185	0.132	0.001	0.017	0.310	0.233
manta	3495	1364	145	15	0.960	0.989	0.094	0.037	0.002	0.011	0.170	0.072
gatk	4391	3225	200	274	0.956	0.922	0.117	0.088	0.022	0.011	0.209	0.161
strelka	2275	1977	40	214	0.983	0.902	0.061	0.054	0.002	0.019	0.115	0.102
delly	5209	537	852	4	0.859	0.993	0.139	0.015	0.003	0.000	0.240	0.029
lumpy	2793		845		0.768		0.075		0.003		0.136	

Table S3. 20× Illumina aligned with bwa mem, All-regions benchmark.

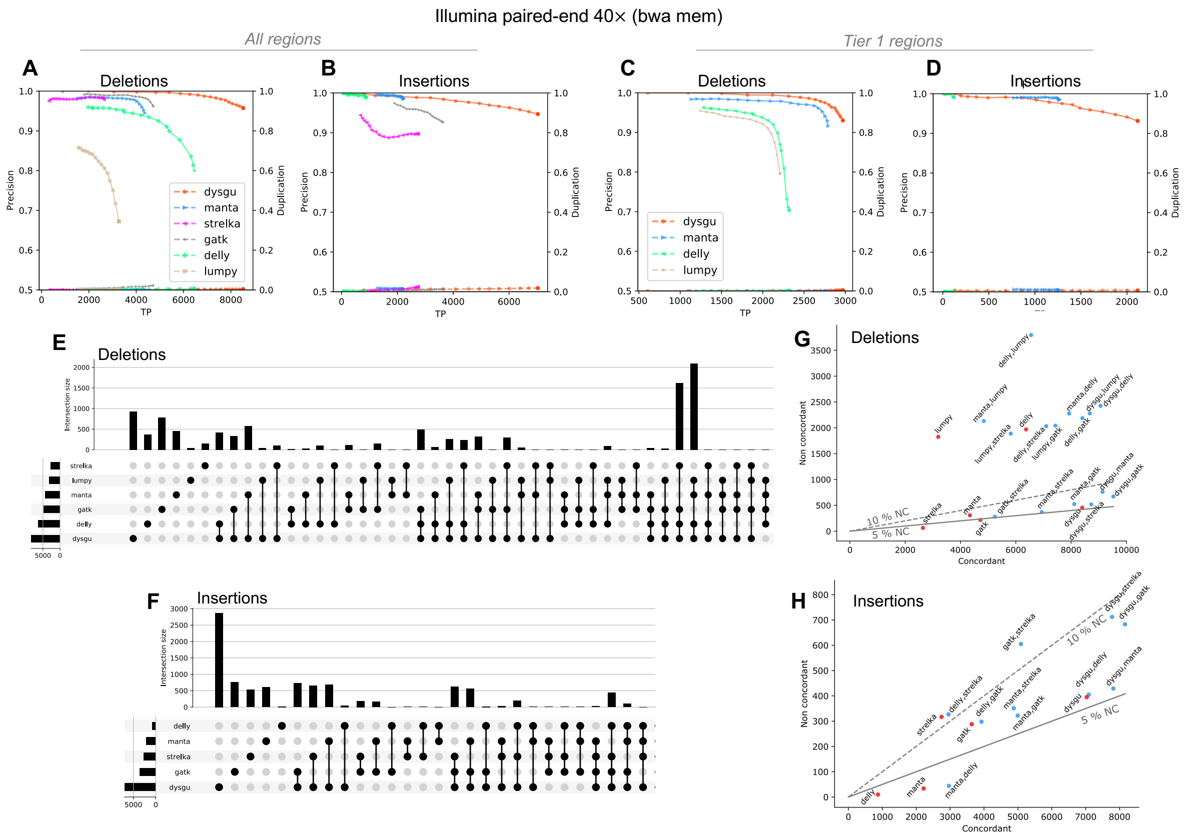

Figure S2. Performance of dysgu using 40× PE reads.
Precision-recall curves are illustrated for all genomic regions (A, B), and high-confidence Tier 1 regions (C, D). A secondary y-axis indicates duplicate true-positives (TP) as a fraction of true-positive calls. Intersections and aggregates of intersections of SV calls for the all-regions benchmark are shown as an upset plot (E, F). Combinations of callers are shown by plotting the union of true-positives between callers (labelled concordant), against the sum of false-positives (labelled non concordant) (G, H). The 5 and 10 % non-concordance (NC) is shown as a solid or dashed line, respectively.

	TP		FP		Precision		Recall		Duplication		F1	
	DEL	INS	DEL	INS	DEL	INS	DEL	INS	DEL	INS	DEL	INS
dysgu	2970	2116	223	156	0.930	0.931	0.704	0.389	0.004	0.007	0.802	0.549
manta	2790	1267	256	20	0.916	0.985	0.662	0.233	0.000	0.009	0.768	0.377
delly	2315	112	978	1	0.703	0.991	0.549	0.021	0.003	0.000	0.617	0.040
lumpy	2210		567		0.796		0.524		0.002		0.632	

Table S4. 40× Illumina aligned with bwa mem, Tier1 benchmark.

	TP		FP		Precision		Recall		Duplication		F1	
	DEL	INS	DEL	INS	DEL	INS	DEL	INS	DEL	INS	DEL	INS
dysgu	8547	7031	379	395	0.957	0.947	0.229	0.192	0.004	0.018	0.369	0.320
manta	4363	2220	255	34	0.945	0.985	0.117	0.061	0.003	0.014	0.208	0.114
gatk	4727	3638	191	288	0.961	0.927	0.126	0.100	0.022	0.014	0.223	0.180
strelka	2642	2747	58	317	0.979	0.897	0.071	0.075	0.002	0.024	0.132	0.139
delly	6460	872	1659	10	0.796	0.989	0.173	0.024	0.006	0.000	0.284	0.047
lumpy	3267		1592		0.672		0.087		0.005		0.155	

Table S5. 40× Illumina aligned with bwa mem, All-regions benchmark.

		Precision				Recall				F1			
		[30, 50)	[50, 500)	[500, 5000)	≥5000	[30, 50)	[50, 500)	[500, 5000)	≥5000	[30, 50)	[50, 500)	[500, 5000)	≥5000
Deletions	dysgu	0.955	0.952	0.974	0.949	0.444	0.288	0.405	0.339	0.606	0.442	0.572	0.500
	manta	1.000	0.948	0.960	0.807	0.013	0.279	0.325	0.360	0.026	0.431	0.486	0.498
	gatk	0.970	0.939	1.000		0.386	0.115	0.001		0.552	0.205	0.003	
	strelka	0.978	1.000			0.304	0.003			0.464	0.005		
	delly	0.962	0.800	0.671	0.266	0.335	0.189	0.409	0.417	0.497	0.305	0.509	0.325
	lumpy	0.706	0.861	0.646	0.216	0.004	0.178	0.414	0.429	0.009	0.295	0.505	0.287
Insertions	dysgu	0.957	0.914	0.995	1.000	0.375	0.238	0.177	0.241	0.539	0.378	0.300	0.389
	manta	0.977	0.984	1.000	1.000	0.017	0.163	0.014	0.004	0.034	0.279	0.028	0.008
	gatk	0.944	0.895	1.000	1.000	0.270	0.121	0.017	0.028	0.420	0.213	0.033	0.054
	strelka	0.882	0.926	1.000		0.311	0.009	0.005		0.460	0.019	0.009	
	delly	0.986	0.993	1.000		0.088	0.011	0.000		0.161	0.022	0.001	

Table S6. 40× Illumina aligned with bwa mem, All-regions benchmark, split by size.

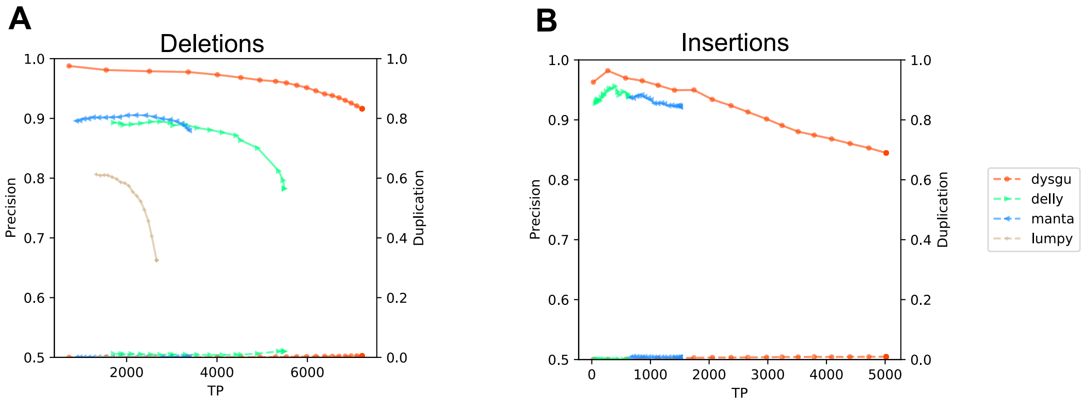

Figure S3. Precision and recall of Syndip benchmark, Illumina reads 45×.

	TP		FP		Precision		Recall		Duplication		F1	
	DEL	INS	DEL	INS	DEL	INS	DEL	INS	DEL	INS	DEL	INS
dysgu	7211	5018	660	922	0.916	0.845	0.142	0.099	0.005	0.009	0.246	0.177
manta	3388	1511	463	128	0.880	0.922	0.067	0.030	0.002	0.007	0.124	0.058
delly	5488	634	1526	41	0.782	0.939	0.108	0.013	0.020	0.000	0.190	0.025
lumpy	2664		1357		0.663		0.053		0.002		0.097	

Table S7. Syndip benchmark results, Illumina reads 45×.

		Precision				Recall				F1			
		[30, 50)	[50, 500)	[500, 5000)	≥5000	[30, 50)	[50, 500)	[500, 5000)	≥5000	[30, 50)	[50, 500)	[500, 5000)	≥5000
Deletions	dysgu	0.931	0.925	0.799	0.852	0.268	0.170	0.262	0.485	0.416	0.287	0.394	0.618
	manta	1.000	0.910	0.759	0.749	0.003	0.147	0.225	0.460	0.006	0.253	0.347	0.569
	delly	0.908	0.789	0.521	0.453	0.217	0.112	0.272	0.500	0.350	0.196	0.358	0.476
	lumpy	0.450	0.822	0.479	0.326	0.001	0.104	0.271	0.500	0.003	0.184	0.346	0.395
Insertions	dysgu	0.876	0.784	1.000	1.000	0.152	0.130	0.232	0.217	0.259	0.222	0.376	0.356
	manta	0.767	0.923	1.000	1.000	0.002	0.079	0.008	0.004	0.004	0.145	0.017	0.007
	delly	0.930	0.971			0.040	0.007	0.000	0.000	0.077	0.014		

Table S8. Syndip benchmark, Illumina reads 45×, split by size.

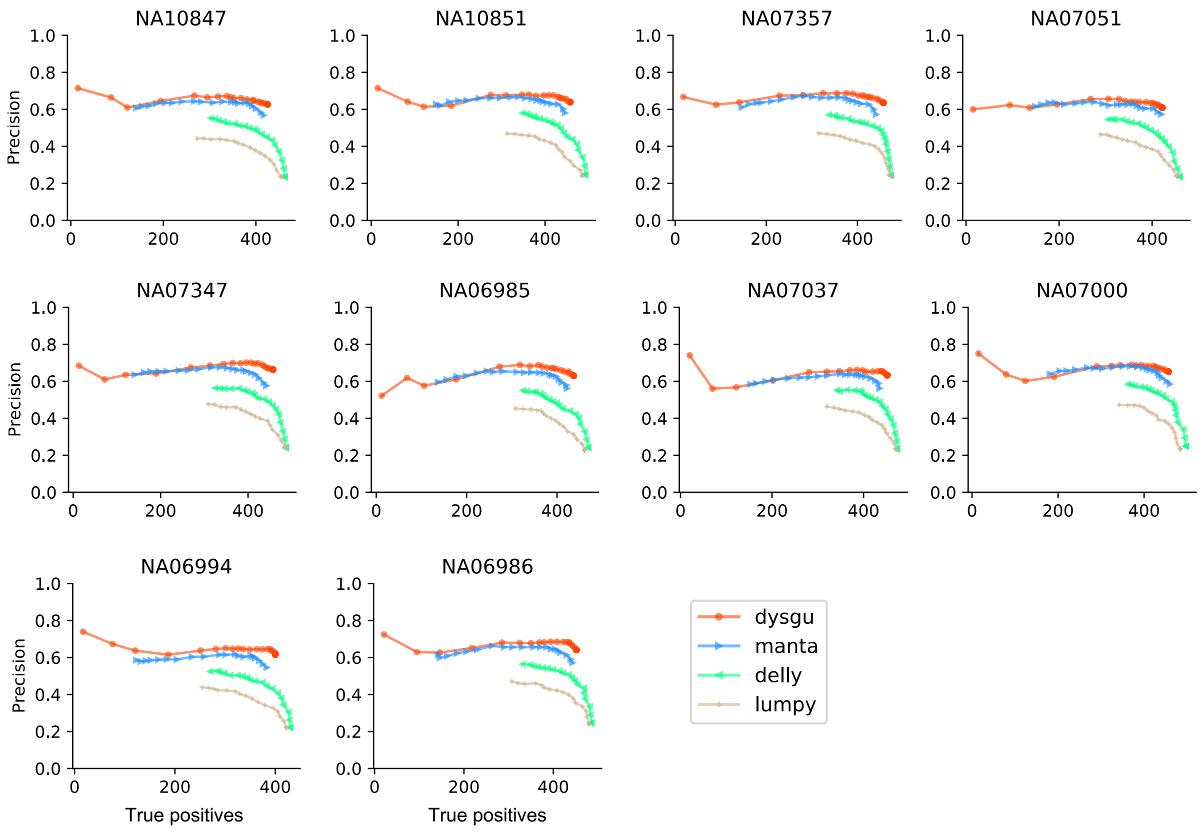

Figure S4. 1000 Genomes benchmark, deletions ≥ 1000 bp.

Sample	Dysgu			Manta			Delly			Lumpy		
	Precision	Recall	F1	Precision	Recall	F1	Precision	Recall	F1	Precision	Recall	F1
NA06985	0.629	0.728	0.675	0.561	0.705	0.625	0.241	0.781	0.368	0.227	0.766	0.350
NA06986	0.637	0.323	0.428	0.570	0.317	0.407	0.244	0.346	0.286	0.241	0.343	0.284
NA06994	0.615	0.295	0.399	0.546	0.283	0.373	0.221	0.316	0.260	0.220	0.311	0.258
NA07000	0.649	0.748	0.695	0.581	0.752	0.655	0.246	0.814	0.378	0.233	0.794	0.360
NA07037	0.633	0.761	0.691	0.560	0.731	0.634	0.233	0.795	0.360	0.231	0.793	0.357
NA07051	0.609	0.314	0.414	0.573	0.313	0.404	0.235	0.341	0.278	0.239	0.336	0.279
NA07347	0.660	0.325	0.436	0.574	0.315	0.407	0.238	0.346	0.282	0.243	0.346	0.285
NA07357	0.634	0.326	0.430	0.569	0.313	0.404	0.242	0.336	0.281	0.239	0.336	0.279
NA10847	0.624	0.742	0.678	0.567	0.732	0.639	0.233	0.807	0.361	0.235	0.797	0.363
NA10851	0.637	0.325	0.431	0.579	0.317	0.410	0.244	0.349	0.287	0.240	0.345	0.283
Mean	0.633	0.489	0.528	0.568	0.478	0.496	0.237	0.523	0.314	0.235	0.517	0.310
stdev	0.015	0.221	0.136	0.010	0.217	0.123	0.007	0.238	0.046	0.007	0.233	0.042

Table S9. 1000 Genomes benchmark, deletions ≥ 1000 bp.

PacBio Sequel II reads

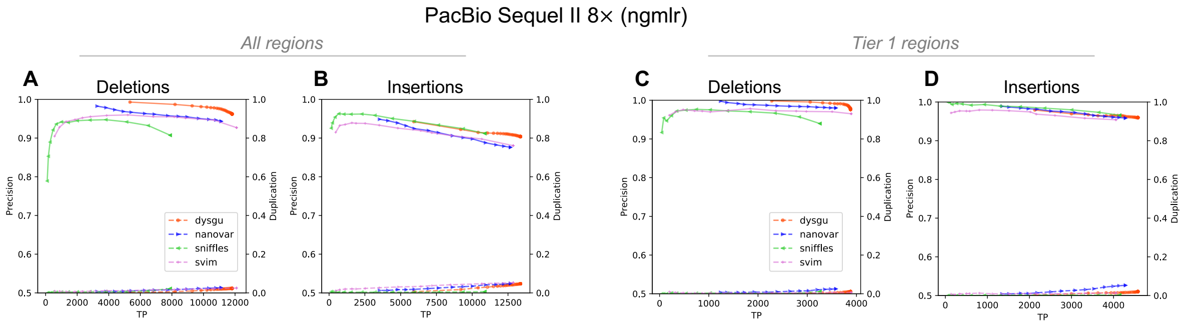

Figure S5. Performance of dysgu on 8× PacBio reads aligned using ngmlr. Precision-recall curves are shown for the 'all-regions' benchmark (A, B), as well as Tier 1 high-confidence regions (C, D).

	TP		FP		Precision		Recall		Duplication		F1	
	DEL	INS	DEL	INS	DEL	INS	DEL	INS	DEL	INS	DEL	INS
dysgu	3882	4588	94	192	0.976	0.960	0.921	0.841	0.009	0.020	0.948	0.898
nanovar	3583	4286	73	186	0.980	0.958	0.850	0.787	0.025	0.053	0.910	0.865
svim	3893	4057	141	195	0.965	0.954	0.923	0.745	0.007	0.013	0.944	0.837
sniffles	3257	4146	209	147	0.940	0.966	0.772	0.762	0.011	0.002	0.848	0.852

Table S10. 8X PacBio Sequel II reads 8× aligned using ngmlr, Tier1 benchmark.

	TP		FP		Precision		Recall		Duplication		F1	
	DEL	INS	DEL	INS	DEL	INS	DEL	INS	DEL	INS	DEL	INS
dysgu	12180	14690	823	2050	0.937	0.878	0.326	0.403	0.029	0.097	0.483	0.551
nanovar	11268	13585	984	2072	0.920	0.868	0.301	0.372	0.024	0.064	0.454	0.520
svim	12487	14942	2456	3584	0.836	0.807	0.334	0.409	0.047	0.142	0.477	0.542
sniffles	7923	12309	1050	1658	0.883	0.881	0.212	0.337	0.027	0.018	0.342	0.487

Table S11. 8× PacBio Sequel II aligned using minimap2, All-regions benchmark.

	TP		FP		Precision		Recall		Duplication		F1	
	DEL	INS	DEL	INS	DEL	INS	DEL	INS	DEL	INS	DEL	INS
dysgu	11810	13372	465	1421	0.962	0.904	0.316	0.366	0.017	0.043	0.476	0.521
nanovar	11124	12675	669	1793	0.943	0.876	0.298	0.347	0.027	0.049	0.452	0.497
svim	12098	12867	955	1748	0.927	0.880	0.324	0.352	0.025	0.053	0.480	0.503
sniffles	7889	10880	806	1052	0.907	0.912	0.211	0.298	0.022	0.006	0.342	0.449

Table S12. 8× PacBio Sequel II aligned using ngmlr, All-regions benchmark.

		Precision				Recall				F1			
		[30, 50)	[50, 500)	[500, 5000)	≥5000	[30, 50)	[50, 500)	[500, 5000)	≥5000	[30, 50)	[50, 500)	[500, 5000)	≥5000
Deletions	dysgu	0.954	0.968	0.965	0.854	0.517	0.484	0.458	0.348	0.670	0.645	0.621	0.494
	nanovar	0.940	0.957	0.915	0.659	0.472	0.450	0.426	0.348	0.629	0.612	0.582	0.455
	svim	0.928	0.936	0.875	0.726	0.526	0.494	0.473	0.374	0.671	0.647	0.614	0.494
	sniffles	0.942	0.951	0.813	0.463	0.257	0.352	0.456	0.419	0.404	0.513	0.585	0.440
Insertions	dysgu	0.859	0.904	0.965	0.917	0.549	0.550	0.404	0.217	0.670	0.684	0.569	0.351
	nanovar	0.853	0.891	0.887	0.352	0.495	0.502	0.459	0.494	0.626	0.642	0.605	0.411
	svim	0.838	0.873	0.968	1.000	0.560	0.524	0.351	0.079	0.671	0.655	0.515	0.147
	sniffles	0.879	0.909	0.968	0.783	0.434	0.475	0.301	0.142	0.581	0.624	0.459	0.241

Table S13. 8× PacBio Sequel II reads aligned with ngmlr, All-regions benchmark, split by size.

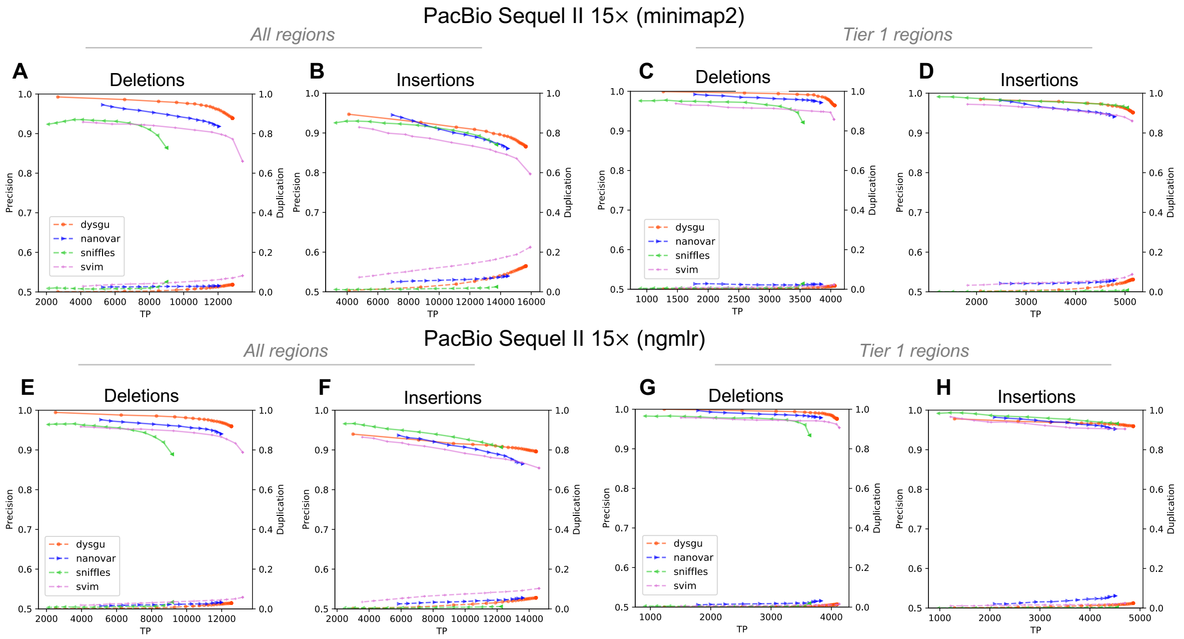

Figure S6. Performance of dysgu on PacBio reads at 15× coverage.

PacBio Sequel II reads were mapped using minimap2 (A – D) and ngmlr (E – H). Precision-recall curves are shown for deletion and insertion events using the ‘all-regions’ benchmark (A,B,E,F) and Tier 1 high-confidence regions (C,D,G,H).

	TP		FP		Precision		Recall		Duplication		F1	
	DEL	INS	DEL	INS	DEL	INS	DEL	INS	DEL	INS	DEL	INS
dysgu	4069	5165	149	258	0.965	0.952	0.965	0.952	0.015	0.053	0.965	0.951
nanovar	3845	4786	113	300	0.972	0.941	0.912	0.879	0.025	0.057	0.941	0.909
svim	4054	5143	310	386	0.929	0.930	0.961	0.945	0.023	0.088	0.945	0.938
sniffles	3544	5043	302	189	0.922	0.964	0.840	0.927	0.029	0.008	0.879	0.945

Table S14. 15× PacBio Sequel II reads aligned using minimap2, Tier1 benchmark.

	TP		FP		Precision		Recall		Duplication		F1	
	DEL	INS	DEL	INS	DEL	INS	DEL	INS	DEL	INS	DEL	INS
dysgu	4093	4865	102	206	0.976	0.959	0.971	0.894	0.014	0.024	0.973	0.925
nanovar	3828	4508	84	225	0.979	0.953	0.908	0.828	0.032	0.061	0.942	0.886
svim	4131	4700	202	235	0.953	0.952	0.980	0.864	0.015	0.024	0.966	0.906
sniffles	3638	4541	257	160	0.934	0.966	0.863	0.834	0.020	0.004	0.897	0.895

Table S15. 15× PacBio Sequel II reads aligned using ngmlr, Tier1 benchmark.

	TP		FP		Precision		Recall		Duplication		F1	
	DEL	INS	DEL	INS	DEL	INS	DEL	INS	DEL	INS	DEL	INS
dysgu	12821	15599	837	2377	0.939	0.868	0.343	0.427	0.028	0.110	0.502	0.572
nanovar	12059	14475	1075	2341	0.918	0.861	0.323	0.396	0.031	0.079	0.477	0.542
svim	13430	15932	2750	4062	0.830	0.797	0.359	0.436	0.082	0.225	0.501	0.563
sniffles	8998	13728	1415	2041	0.864	0.871	0.241	0.375	0.051	0.025	0.377	0.525

Table S16. 15× PacBio Sequel II reads aligned using minimap2, All-regions benchmark.

	TP		FP		Precision		Recall		Duplication		F1	
	DEL	INS	DEL	INS	DEL	INS	DEL	INS	DEL	INS	DEL	INS
dysgu	12555	14429	529	1671	0.960	0.896	0.336	0.395	0.029	0.056	0.498	0.548
nanovar	11982	13608	759	2119	0.940	0.865	0.321	0.372	0.033	0.055	0.478	0.520
svim	13203	14618	1566	2491	0.894	0.854	0.353	0.400	0.058	0.103	0.506	0.545
sniffles	9175	12245	1144	1256	0.889	0.907	0.245	0.335	0.034	0.012	0.385	0.489

Table S17. 15× PacBio Sequel II reads aligned using ngmlr, All-regions benchmark.

		Precision				Recall				F1			
		[30, 50)	[50, 500)	[500, 5000)	≥5000	[30, 50)	[50, 500)	[500, 5000)	≥5000	[30, 50)	[50, 500)	[500, 5000)	≥5000
Deletions	dysgu	0.934	0.937	0.948	0.923	0.581	0.531	0.447	0.319	0.716	0.678	0.608	0.474
	nanovar	0.926	0.917	0.869	0.795	0.523	0.490	0.464	0.342	0.669	0.639	0.605	0.478
	svim	0.851	0.799	0.851	0.919	0.604	0.553	0.483	0.301	0.706	0.654	0.616	0.453
	sniffles	0.917	0.877	0.764	0.497	0.310	0.407	0.475	0.384	0.463	0.556	0.586	0.434
Insertions	dysgu	0.817	0.855	0.949	0.892	0.623	0.635	0.552	0.261	0.707	0.729	0.698	0.404
	nanovar	0.836	0.860	0.835	0.374	0.551	0.590	0.528	0.134	0.665	0.700	0.647	0.198
	svim	0.763	0.761	0.907	0.970	0.631	0.648	0.580	0.257	0.691	0.700	0.707	0.406
	sniffles	0.849	0.858	0.893	0.853	0.504	0.588	0.535	0.253	0.633	0.698	0.670	0.390

Table S18. 15× PacBio Sequel II reads aligned with minimap2, All-regions benchmark, split by size.

		Precision				Recall				F1			
		[30, 50)	[50, 500)	[500, 5000)	≥5000	[30, 50)	[50, 500)	[500, 5000)	≥5000	[30, 50)	[50, 500)	[500, 5000)	≥5000
Deletions	dysgu	0.948	0.967	0.962	0.865	0.546	0.513	0.484	0.327	0.693	0.670	0.644	0.475
	nanovar	0.938	0.950	0.920	0.659	0.507	0.482	0.462	0.360	0.658	0.640	0.615	0.466
	svim	0.900	0.905	0.815	0.625	0.569	0.534	0.525	0.403	0.697	0.672	0.639	0.490
	sniffles	0.943	0.949	0.760	0.355	0.307	0.410	0.495	0.454	0.463	0.573	0.599	0.399
Insertions	dysgu	0.843	0.895	0.975	0.939	0.587	0.588	0.438	0.182	0.692	0.709	0.604	0.305
	nanovar	0.846	0.876	0.876	0.317	0.532	0.531	0.485	0.510	0.653	0.661	0.624	0.391
	svim	0.804	0.840	0.950	1.000	0.600	0.589	0.444	0.126	0.687	0.693	0.606	0.225
	sniffles	0.865	0.907	0.967	0.774	0.482	0.520	0.366	0.285	0.619	0.661	0.531	0.416

Table S19. 15× PacBio Sequel II reads aligned with ngmlr, All-regions benchmark, split by size.

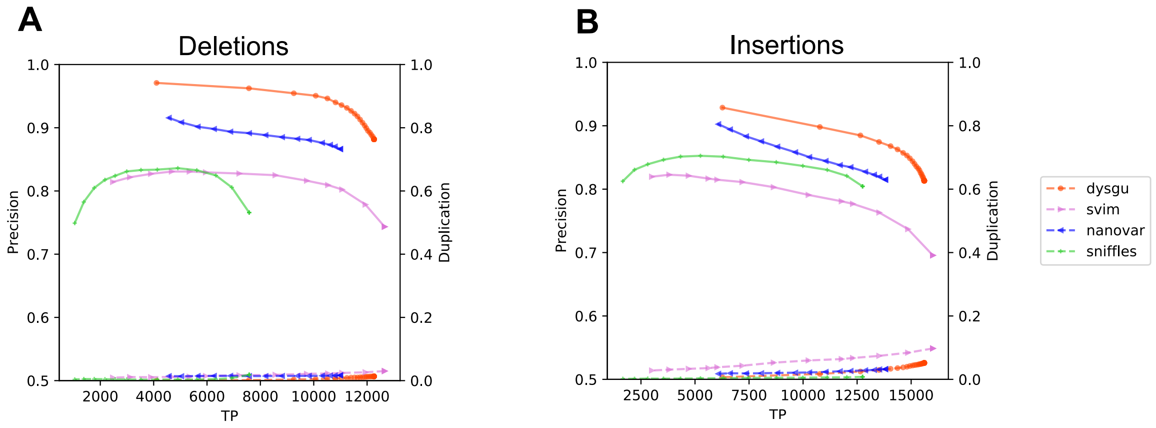

Figure S7. Precision and recall of Syndip benchmark, PacBio reads 12×.

	TP		FP		Precision		Recall		Duplication		F1	
	DEL	INS	DEL	INS	DEL	INS	DEL	INS	DEL	INS	DEL	INS
dysgu	12260	15606	1645	3582	0.882	0.813	0.242	0.308	0.013	0.052	0.380	0.447
nanovar	10995	13807	1697	3136	0.866	0.815	0.217	0.272	0.017	0.032	0.347	0.408
svim	12657	16004	4368	7003	0.743	0.696	0.250	0.316	0.030	0.098	0.374	0.434
sniffles	7580	12753	2318	3100	0.766	0.805	0.150	0.252	0.019	0.008	0.250	0.383

Table S20. Syndip benchmark results, PacBio reads 12×.

		Precision				Recall				F1			
		[30, 50)	[50, 500)	[500, 5000)	≥5000	[30, 50)	[50, 500)	[500, 5000)	≥5000	[30, 50)	[50, 500)	[500, 5000)	≥5000
Deletions	dysgu	0.918	0.872	0.723	0.873	0.397	0.346	0.310	0.529	0.554	0.496	0.434	0.659
	nanovar	0.915	0.872	0.597	0.734	0.359	0.307	0.277	0.496	0.516	0.454	0.378	0.592
	svim	0.808	0.711	0.601	0.842	0.401	0.362	0.318	0.529	0.536	0.480	0.416	0.650
	sniffles	0.862	0.812	0.489	0.371	0.171	0.246	0.305	0.526	0.285	0.377	0.376	0.435
Insertions	dysgu	0.798	0.838	0.655	0.788	0.382	0.480	0.583	0.327	0.517	0.610	0.617	0.462
	nanovar	0.821	0.859	0.540	0.466	0.334	0.424	0.487	0.202	0.475	0.568	0.512	0.282
	svim	0.694	0.696	0.587	0.776	0.391	0.497	0.574	0.279	0.500	0.580	0.580	0.411
	sniffles	0.810	0.832	0.620	0.705	0.288	0.408	0.545	0.272	0.425	0.547	0.580	0.393

Table S21. Syndip benchmark, PacBio reads 12×, split by size.

ONT reads

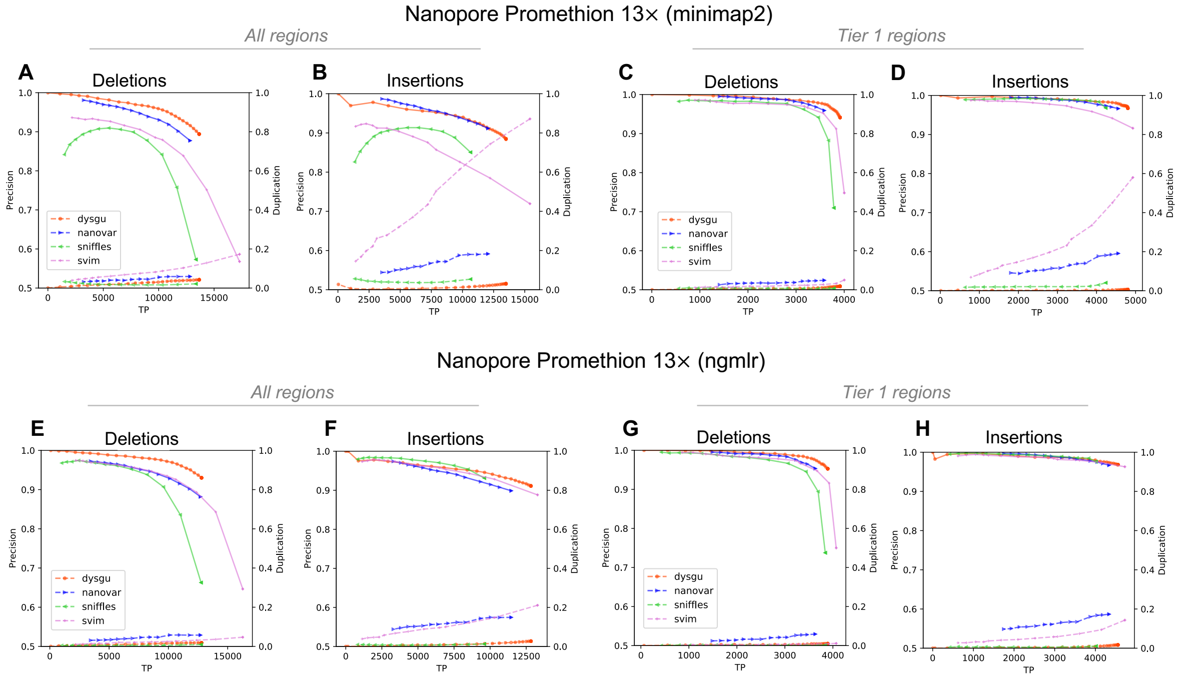

Figure S8. Performance of dysgu using ONT reads.

ONT reads were mapped using minimap2 (A – D) and ngmlr (E – H). Precision-recall curves are shown for deletion and insertion events using the 'all-regions' benchmark (A,B,E,F) and Tier 1 high-confidence regions (C,D,G,H).

	TP		FP		Precision		Recall		Duplication		F1	
	DEL	INS	DEL	INS	DEL	INS	DEL	INS	DEL	INS	DEL	INS
dysgu	3918	4803	247	168	0.941	0.966	0.921	0.882	0.017	0.005	0.935	0.922
nanovar	3587	4556	153	159	0.959	0.966	0.851	0.837	0.049	0.191	0.902	0.897
svim	4002	4929	1348	452	0.748	0.916	0.949	0.906	0.050	0.580	0.837	0.911
sniffles	3785	4236	1548	140	0.710	0.968	0.898	0.778	0.007	0.041	0.793	0.863

Table S22. 13× ONT Promethion reads aligned using minimap2, Tier1 benchmark.

	TP		FP		Precision		Recall		Duplication		F1	
	DEL	INS	DEL	INS	DEL	INS	DEL	INS	DEL	INS	DEL	INS
dysgu	3893	4555	193	149	0.953	0.968	0.923	0.837	0.010	0.016	0.938	0.898
nanovar	3634	4337	178	148	0.953	0.967	0.862	0.797	0.059	0.173	0.905	0.874
svim	4069	4724	1356	182	0.750	0.963	0.965	0.868	0.012	0.143	0.844	0.913
sniffles	3841	4016	1366	99	0.738	0.976	0.911	0.738	0.004	0.010	0.815	0.840

Table S23. 13× ONT Promethion reads aligned using ngmlr, Tier1 benchmark.

	TP		FP		Precision		Recall		Duplication		F1	
	DEL	INS	DEL	INS	DEL	INS	DEL	INS	DEL	INS	DEL	INS
dysgu	13716	13531	1680	1854	0.8909	0.8846	0.3668	0.369	0.041	0.028	0.5196	0.5209
nanovar	12835	12051	1794	1168	0.8774	0.9116	0.3433	0.3296	0.059	0.183	0.4935	0.4841
svim	17297	15418	13167	6015	0.5678	0.7194	0.4626	0.4216	0.1729	0.8716	0.5098	0.5316
sniffles	13363	10646	9958	1870	0.573	0.8506	0.3574	0.2911	0.0214	0.0537	0.4402	0.4338

Table S24. 13× ONT Promethion reads aligned using minimap2, All-regions benchmark.

	TP		FP		Precision		Recall		Duplication		F1	
	DEL	INS	DEL	INS	DEL	INS	DEL	INS	DEL	INS	DEL	INS
dysgu	12829	12860	1000	1294	0.928	0.909	0.343	0.352	0.018	0.026	0.501	0.507
nanovar	12706	11464	1702	1292	0.882	0.899	0.340	0.314	0.058	0.149	0.491	0.465
svim	16275	13286	8904	1673	0.646	0.888	0.435	0.363	0.047	0.211	0.520	0.516
sniffles	12757	9604	6491	710	0.663	0.931	0.341	0.263	0.010	0.013	0.451	0.410

Table S25. 13× ONT Promethion reads aligned using ngmlr, All-regions benchmark.

		Precision				Recall				F1			
		[30, 50)	[50, 500)	[500, 5000)	≥5000	[30, 50)	[50, 500)	[500, 5000)	≥5000	[30, 50)	[50, 500)	[500, 5000)	≥5000
Deletions	dysgu	0.840	0.922	0.841	0.814	0.535	0.515	0.511	0.339	0.654	0.661	0.636	0.479
	nanovar	0.746	0.945	0.927	0.635	0.460	0.454	0.429	0.337	0.569	0.614	0.587	0.441
	svim	0.346	0.688	0.840	0.924	0.622	0.569	0.499	0.323	0.445	0.623	0.626	0.479
	sniffles	0.357	0.692	0.772	0.439	0.460	0.464	0.483	0.393	0.402	0.556	0.594	0.415
Insertions	dysgu	0.839	0.883	0.920	0.917	0.464	0.592	0.544	0.613	0.597	0.709	0.684	0.735
	nanovar	0.934	0.905	0.891	0.618	0.358	0.544	0.562	0.455	0.517	0.679	0.689	0.524
	svim	0.669	0.668	0.922	0.963	0.524	0.662	0.645	0.510	0.588	0.665	0.759	0.667
	sniffles	0.834	0.829	0.891	0.902	0.328	0.477	0.502	0.474	0.471	0.606	0.642	0.622

Table S26. 13× ONT Promethion reads aligned with minimap2, All-regions benchmark, split by size.

		Precision				Recall				F1			
		[30, 50)	[50, 500)	[500, 5000)	≥5000	[30, 50)	[50, 500)	[500, 5000)	≥5000	[30, 50)	[50, 500)	[500, 5000)	≥5000
Deletions	dysgu	0.882	0.947	0.944	0.928	0.499	0.495	0.476	0.290	0.638	0.650	0.633	0.442
	nanovar	0.781	0.936	0.919	0.494	0.453	0.459	0.436	0.362	0.574	0.616	0.591	0.418
	svim	0.419	0.779	0.893	0.730	0.590	0.541	0.514	0.370	0.490	0.639	0.653	0.491
	sniffles	0.468	0.782	0.782	0.307	0.437	0.453	0.509	0.472	0.452	0.573	0.617	0.372
Insertions	dysgu	0.877	0.905	0.955	1.000	0.442	0.573	0.503	0.174	0.588	0.701	0.659	0.296
	nanovar	0.934	0.900	0.893	0.386	0.337	0.515	0.531	0.565	0.496	0.655	0.666	0.459
	svim	0.862	0.870	0.961	1.000	0.455	0.603	0.488	0.166	0.595	0.712	0.647	0.285
	sniffles	0.913	0.926	0.953	0.870	0.300	0.441	0.412	0.372	0.452	0.598	0.575	0.521

Table S27. 13× ONT Promethion reads aligned with ngmlr, All-regions benchmark, split by size.

Combinations of sequencing platforms

	TP		Precision		Recall		Duplication		F1	
	DEL	INS	DEL	INS	DEL	INS	DEL	INS	DEL	INS
pb 8x	3898	4959	0.967	0.959	0.924	0.911	0.014	0.040	0.945	0.934
pb 8x + ill 20x	4031	4988	0.945	0.948	0.956	0.916	0.035	0.051	0.951	0.932
pb 8x + ill 40x	4048	4992	0.923	0.936	0.960	0.917	0.050	0.063	0.941	0.926
pb 15x	4069	5165	0.965	0.952	0.965	0.949	0.015	0.053	0.965	0.951
pb 15x + ill 20x	4106	5185	0.943	0.942	0.974	0.953	0.037	0.064	0.958	0.947
pb 15x + ill 40x	4108	5169	0.920	0.930	0.974	0.950	0.054	0.076	0.946	0.940
ont 13x	3918	4803	0.941	0.966	0.929	0.882	0.017	0.005	0.935	0.922
ont 13x ill 20x	4005	4846	0.920	0.937	0.950	0.890	0.030	0.125	0.935	0.913
ont 13x ill 40x	4029	4918	0.875	0.902	0.955	0.904	0.039	0.194	0.913	0.903
ont 13x pb 8x	4117	5086	0.937	0.945	0.976	0.934	0.054	0.359	0.956	0.940

Table S28. Combinations of sequencing platforms, Tier1 benchmark.
Calls were made using dysgu on each dataset before merging of outputs. Calls were tested using Tier1 regions on the HG002 benchmark. The coverage value of each sequencing dataset is denoted using 'x'. pb – PacBio Sequel II, ill – Illumina 150bp PE, ont – Oxford Nanopore Technologies Promethion.

	TP		Precision		Recall		Duplication		F1	
	DEL	INS	DEL	INS	DEL	INS	DEL	INS	DEL	INS
pb 8x	8599	11158	0.953	0.926	0.443	0.524	0.039	0.119	0.605	0.669
pb 8x + ill 20x	9075	11434	0.951	0.924	0.467	0.537	0.058	0.140	0.627	0.679
pb 8x + ill 40x	9297	11604	0.947	0.920	0.479	0.545	0.071	0.153	0.636	0.684
pb 15x	9009	11693	0.960	0.921	0.464	0.549	0.039	0.136	0.626	0.688
pb 15x + ill 20x	9352	11899	0.957	0.919	0.482	0.559	0.058	0.158	0.641	0.695
pb 15x + ill 40x	9518	12015	0.953	0.915	0.490	0.564	0.073	0.173	0.647	0.698
ont 13x	9221	10668	0.949	0.924	0.475	0.501	0.059	0.032	0.633	0.650
ont 13x ill 20x	9170	10998	0.953	0.922	0.472	0.516	0.060	0.124	0.632	0.662
ont 13x ill 40x	9475	11285	0.947	0.914	0.488	0.530	0.075	0.173	0.644	0.671
ont 13x pb 8x	9679	11784	0.921	0.902	0.498	0.553	0.113	0.280	0.647	0.686

Table S29. Combinations of sequencing platforms, Tier1+2 benchmark.
Calls were made using dysgu on each dataset before merging of outputs. Calls were tested using Tier1+2 regions on the HG002 benchmark. The coverage value of each sequencing dataset is denoted using 'x'. pb – PacBio Sequel II, ill – Illumina 150bp PE, ont – Oxford Nanopore Technologies Promethion.

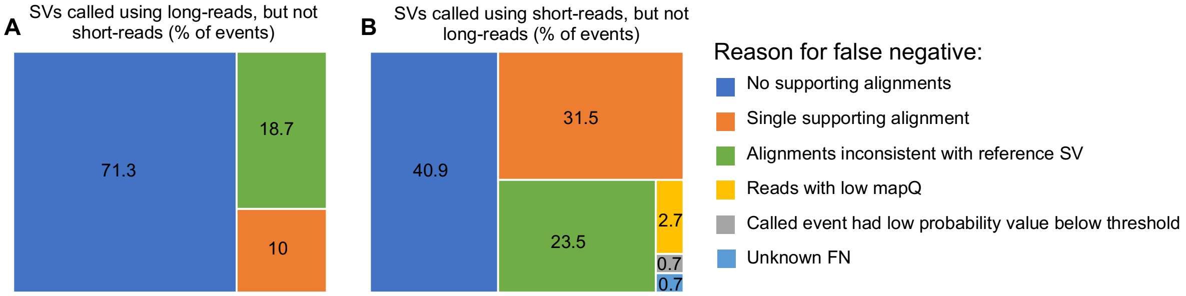

Figure S9. Differences in deletion calls between short and long reads using dysgu. Deletion SVs were called using 20× PE reads (aligned using bwa mem) and 8× PacBio reads (aligned using minimap2). For each platform, 150 true positive calls were analysed that were absent from the other platform. SVs called using PE reads only are shown in A, and SVs called only using LRs are shown in B. The reasons that events were absent from the other platform was determined by manually inspecting the alignment files. Events missed due to "No supporting alignments" or "Single supporting alignment" could conceivably by detected if sequencing depth was increased. Events labelled with "Reads with low mapQ" or "Called event had low probability value below threshold" would be detected by altering the default parameters of dysgu. Events labelled "Unknown FN" appeared to have sufficient evidence but lacked a call from dysgu, suggesting an opportunity for future improvements. Events labelled "Alignments inconsistent with reference SV" had reads mapped to the correct location that appeared to support the SV but had an alternative alignment representation that did not match the reference call. Additionally, this category also included events with soft-clipped reads that appeared to support the SV, but no spanning or supplementary alignments. For this category, alternative alignment or analysis approaches may be required for detection.

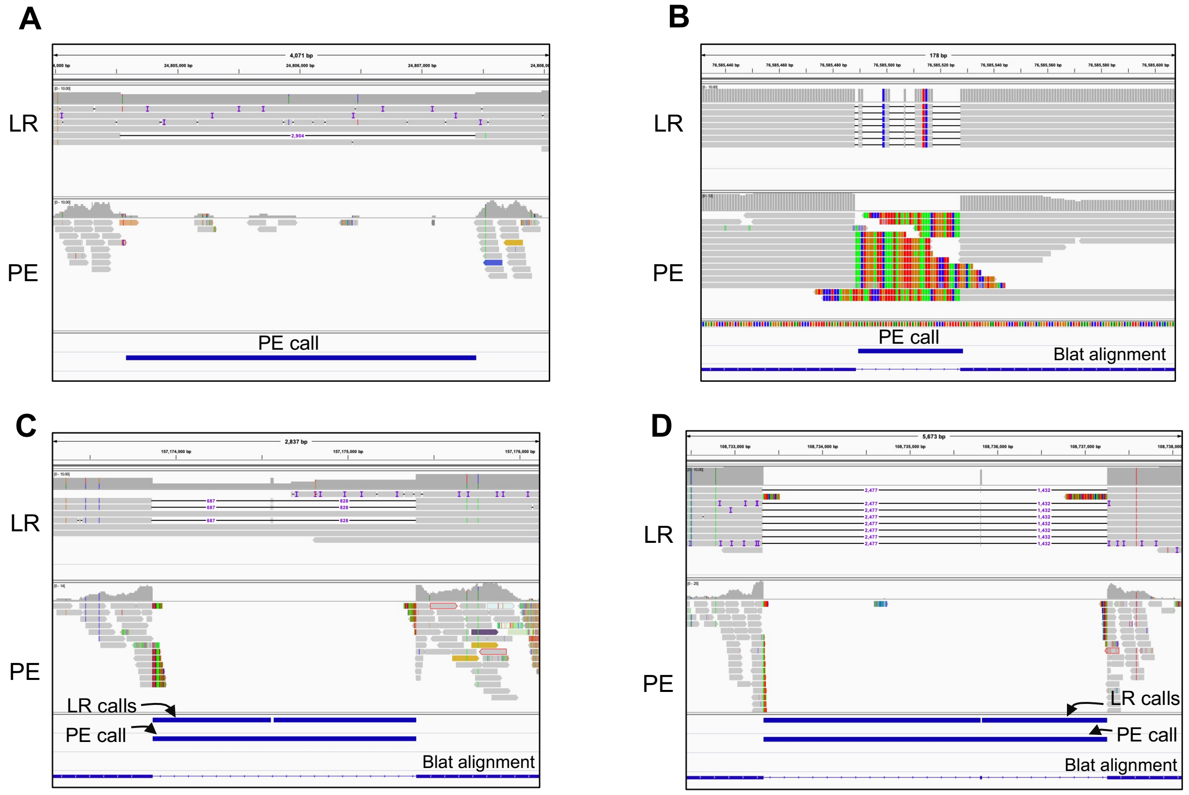

Figure S10. Examples of deletion SVs called correctly using PE reads but not LR.

Deletion SVs were called using 20× PE reads (aligned using bwa mem) and 8× PacBio reads (LR) (aligned using minimap2). Examples are given of SVs that were correctly called using PE reads but not LR. In A, only a single alignment is present in LR data which is below the detection threshold. In example B, LR alignments are inconsistent with the reference SV, however alignment of reads using blat identified the expected reference SV. In example C, dysgu called two SVs using LR data, although only one SV is called using PE data. Aligning LRs using blat however recovered the expected deletion. In example D dysgu again called two SVs using LR when a single longer deletion was expected. However, in this case blat did not recover the single-gap deletion alignment.

	Coverage	Platform	Read length	Mins	Memory (GB)	Space (GB)
HG005	16	Illumina	250	38.5	3.6	7.4
NA12878	17	BGI	100	22.5	1.9	2.3
NA12878	31	Illumina	148	42.0	3.8	5.8
NA07357	31	Illumina	150	51.2	4.0	8.5
NA06986	32	Illumina	150	58.4	4.5	9.4
NA06994	32	Illumina	150	67.9	4.8	9.6
NA06985	35	Illumina	150	71.6	4.9	9.8
CHM1-CHM13	45	Illumina	151	105.4	12.8	25
HG00512	76	Illumina	126	130.5	9.4	13.8

Table S30. Resource requirements of dysgu using PE data.
